# Seizures in Fragile X Syndrome: Associations and Longitudinal Analysis of a Large Clinic-Based Cohort

**DOI:** 10.3389/fped.2021.736255

**Published:** 2021-12-30

**Authors:** Elizabeth Berry-Kravis, Robyn A. Filipink, Richard E. Frye, Sailaja Golla, Stephanie M. Morris, Howard Andrews, Tse-Hwei Choo, Walter E. Kaufmann, The FORWARD Consortium, Elizabeth Berry-Kravis

**Affiliations:** ^1^Department of Pediatrics, Rush University Medical Center, Chicago, IL, United States; ^2^Department of Neurological Sciences, Rush University Medical Center, Chicago, IL, United States; ^3^Department of Pediatrics, University of Pittsburgh School of Medicine, Pittsburgh, PA, United States; ^4^Barrow Neurological Institute at Phoenix Children's Hospital, Phoenix, AZ, United States; ^5^Department of Child Health, University of Arizona College of Medicine–Phoenix, Phoenix, AZ, United States; ^6^Division of Neurodevelopmental Medicine, Department of Neurology, Thompson Autism Center, Children's Hospital of California, University of Irvine, Orange, CA, United States; ^7^Division of Pediatric and Developmental Neurology, Department of Neurology, Washington University in St. Louis, St. Louis, MO, United States; ^8^Department of Biostatistics, Mailman School of Public Health, Columbia University Medical Center, New York, NY, United States; ^9^Department of Psychiatry, New York State Psychiatric Institute, Columbia University Medical Center, New York, NY, United States; ^10^Emory University School of Medicine, Atlanta, GA, United States

**Keywords:** Fragile X syndrome, seizures, epilepsy, longitudinal, autism spectrum disorder

## Abstract

Fragile X syndrome (FXS), the most common inherited cause of intellectual disability, learning disability, and autism spectrum disorder, is associated with an increased prevalence of certain medical conditions including seizures. The goal of this study was to better understand seizures in individuals with FXS using the Fragile X Online Registry with Accessible Research Database, a multisite observational study initiated in 2012 involving FXS clinics in the Fragile X Clinic and Research Consortium. Seizure data were available for 1,607 participants, mostly male (77%) and white (74.5%). The overall prevalence of at least one seizure was 12%, with this rate being significantly higher in males than females (13.7 vs. 6.2%, *p* < 0.001). As compared to individuals with FXS without seizures, those with seizures were more likely to have autism spectrum disorder, current sleep apnea, later acquisition of expressive language, more severe intellectual disability, hyperactivity, irritability, and stereotyped movements. The mean age of seizure onset was 6.4 (SD 6.1) years of age with the great majority (>80%) having onset of seizures which was before 10. For those with epilepsy, about half (52%) had seizures for more than 3 years. This group was found to have greater cognitive and language impairment, but not behavioral disruptions, compared with those with seizures for <3 years. Antiepileptic drugs were more often used in males (60.6%) than females (34.8%), and females more often required more than one medication. The most commonly used anticonvulsants were oxcarbazepine, valproic acid, lamotrigine, and levetiracetam. The current study is the largest and first longitudinal study ever conducted to describe seizures in FXS. Overall, this study confirms previous reports of seizures in FXS and extends previous findings by further defining the cognitive and behavioral phenotype of those with epilepsy in FXS. Future studies should further investigate the natural history of seizures in FXS and the characteristics of seizures in FXS in adulthood.

## Introduction

Fragile X syndrome (FXS) is the most common known inherited cause of intellectual disability, learning disability, and autism spectrum disorder (ASD), with an estimated prevalence of about 1/4,000 (1). Individuals with FXS display variable physical features such as large ears, long face, macrocephaly, macroorchidism, and variable levels of functioning, with a fairly stereotyped cognitive profile and behavioral features characterized by hyperactivity, anxiety, and socialization difficulties. Additionally, certain medical problems are more common in FXS, such as strabismus, otitis media, gastroesophageal reflux, loose stools, sleep apnea, and seizures (2).

FXS results from a trinucleotide repeat (CGG) expansion mutation of >200 repeats (full mutation) in the promoter of *FMR1* (*fragile X mental retardation 1*) gene (3) which leads to transcriptional silencing of *FMR1* and loss or significant reduction of expression of the gene product, FMRP (fragile X mental retardation protein) (4). FMRP is an RNA-binding protein that appears to function as a dendritic translational repressor that modulates receptor-activated dendritic protein synthesis and regulates multiple ion channels (5–7). In the absence of FMRP in the *Fmr1* knockout (k/o) mouse model of FXS, there is immature dendritic spine morphology and region- and cell-dependent deficits in synaptic plasticity (8). Accordingly, these synaptic abnormalities result in abnormal epileptiform discharges (9) and a high frequency of audiogenic seizures in the *Fmr1* k/o mouse (10), presumably modeling the increased risk of seizures in humans with FXS.

Past reports have identified seizures in 4.4%-40% of males with FXS (2, 11–19) with a lower frequency of 4.4%-18% in larger cohorts with less referral bias (2, 13, 15–19). Many children with FXS also have abnormal electroencephalograms (EEGs) without overt epileptic seizures (15, 17, 19), frequently with a pattern of centrotemporal spikes (similar to benign focal epilepsy of childhood) (13, 15, 19, 20). Types of seizures reported in FXS vary widely, and clinical and EEG findings consistent with Panayiotopoulos syndrome, a benign autonomic epilepsy syndrome, have also been seen in FXS (21). Complex partial (focal onset with impaired awareness) seizures were reported as most common in FXS in several series (13, 15, 18), although simple partial (focal onset without impaired awareness) and generalized tonic-clonic seizures occur often, and status epilepticus has also been observed (22). Seizures are reported to be easily controlled in most cases (15, 17, 18) and resolve during childhood in the majority of individuals with FXS, although in a study of hospital encounters for adolescents and adults with FXS, seizures represented an identifiable reason for emergency room presentation and hospitalization in FXS (23). Occasional patients with FXS and intractable seizures due to mesial temporal sclerosis have been described, suggesting a secondary etiology amenable to surgical management (24). A small case series describing three females with FXS and severe developmental impairment and medically refractory focal epilepsy also suggests that severe epilepsy in FXS may signal a secondary genetic condition (25).

Studies investigating the association of seizures with other disease features in FXS include a small cohort which showed a trend toward an increased rate of seizures in individuals who were also diagnosed with ASD (26) and a second small case series of 11 patients which suggested an association between epilepsy and attention problems (27). In the largest investigation of seizures in FXS involving 1,394 individuals assessed by a survey and 352 individuals assessed in clinic (19), 14% of males and 6% of females reported seizures. Seizures tended to be infrequent and more often focal, had onset between 4 and 10 years of age, were generally easily treated, and were associated with ASD, but not academic achievement. A clinic-based study of 135 patients with FXS seen in clinic (17) suggested that many patients described spells such as staring, but most of these were without EEG correlate, and only 4.4% of the cohort were actually diagnosed with seizures, emphasizing the importance of clinical confirmation of seizure diagnoses in persons with FXS. A large study of ASD in FXS based on data from 547 participants with FXS in the Fragile X Online Registry and Accessible Research Database (FORWARD) natural history study (28) showed that having a diagnosis of ASD was associated with an approximately three-fold increased risk of seizures both in younger children and in adolescent/young adults (29).

The Fragile X Clinic and Research Consortium (FXCRC) initial database collected data from 260 participants with FXS from 9 FXS Clinics from 2005 to 2011 and showed a seizure frequency of 10% (12% in males) (2). Longitudinal data about seizures from participants with FXS have subsequently been collected as part of the FORWARD project (28), from 2012 to 2021. In the present report, we use these FORWARD data to investigate seizure prevalence and characteristics, including gender differences, age of onset and resolution, duration and associated comorbid conditions, and other features (e.g., use of anticonvulsants), in the largest and first longitudinal study ever conducted to describe seizures in FXS. Specific goals of these analyses are to update information on seizure frequency and co-occurring problems associated with seizures in FXS in a larger group than previously reported, study the onset and trajectory of seizures experienced by those with FXS across time with longitudinal data, and understand factors associated with more severe epilepsy in FXS.

## Materials and Methods

Data analyzed for this report were derived from FORWARD. As described previously (28), FORWARD is a multisite observational study initiated in 2012. The study collects data yearly on a Registry form, Parent Report form, and Clinician Report form, as well as standardized questionnaires including the Aberrant Behavior Checklist-Community Edition (ABC-C) (29), the Social Communication Questionnaire (SCQ) (30), and the Social Responsiveness Scale – Second Edition (SRS-2) (31). The analyses for this report were performed using baseline and longitudinal data collected during follow-up visits from FORWARD Version 5, with data obtained from 1,607 individuals with FXS evaluated between 2012 and 2020, who had information available about seizures derived from the Clinician Report form. There were 1,607 baseline visits and 1,945 follow-up visits (occurring after the baseline visit) for 803 participants with at least 1 year of follow-up. The study was approved by the Institutional Review Board for each participating FXS Clinic where data were collected, and written informed consent was obtained from primary caregivers or adult patients who were their own guardians.

Demographic variables including age, sex, and ethnicity were collected on the Registry form. Data from the Clinician Report form included variables related to seizures including presence or absence of seizures currently or in the past, age (in years and months) at seizure onset, age when seizures resolved if the participant had been 2 years without seizures, type of seizures (focal, generalized, febrile), whether antiepileptic medication was used for seizures at the time of the visit, and, if so, which antiepileptic medication(s) the participant was taking. Antiepileptic medication use was tracked at all visits and was reported in this paper based on use described at any visit over the course of follow-up. Other data from the Clinician Report form about problems that could potentially be associated with seizures were also utilized, including level of intellectual disability (ID), presence of ASD, presence of sleep apnea, severity of behaviors on the ABC-C adjusted for FXS (ABC_FX_) (32), and severity of ASD symptoms on the SCQ and SRS-2.

## Statistical Analysis

Frequency tabulations and proportions for categorical variables, and means and standard deviations for continuous variables, were used for the descriptive analyses. The chi-square test or Fisher's exact test for categorical variables, and analysis of variance (ANOVA) for continuous variables were used to compare characteristics of those with and without seizure experience and to compare males and females with seizures. Using longitudinal data, subjects were divided into three groups based on seizure experience. Participants who reported no seizures ever and who were observed at least once at or over the age of 15 comprised one group; participants who reported having seizures but only for a period <3 years and were followed at least 2 years after the last reported seizure comprised a second group; and participants who reported seizures over a period >3 years comprised the third group. Comparisons of demographic and clinical variables between these three groups were made using chi-square tests or Fisher's exact tests, for categorical variables, and Wilcoxon rank-sum test and Student's *t*-tests for continuous variables, as appropriate.

Kaplan–Meier survival estimates were produced to model and plot time to first reported seizure for the full sample. Mixed-effect logistic regression models were fit to estimate the rate of current seizures for age groups, using the longitudinal data, across the full sample. This model featured random intercepts for subject.

Analyses were performed using SPSS version 26 and SAS version 9.4. In each analysis reported in this paper, data were used for all individuals who had valid values for the variables used in that analysis. Statistical significance level was set at 5%.

## Results

Characteristics of the 1,607 FORWARD participants with available seizure data used for this study are shown in Table 1: 77% were male, 74.5% were White, 7.7% were African American/Black, 3.4% were Asian, and 12.9% were Hispanic. The mean age at the baseline evaluation for the cohort was 13.8 years. A history of current or past seizures at any evaluation was reported for 193 patients (170 males and 23 females) or 12% of the sample. Approximately half had moderate (38.7%) or severe/profound (7.5%) ID, ~39.5% were diagnosed with ASD by a clinician, and sleep apnea was present in 16.9% of the cohort. Severity of ID, ASD symptom severity by SRS-2, and percent of patients with an ASD diagnosis by SCQ were all lower in females with FXS relative to males.

**Table 1:** Demographics of the FORWARD cohort.

Characteristic (*N*)^*^	
Sex (*N* = 1607)	77% male
Race/ethnicity (*N* = 1607)	74.5% White, 7.7% African American, 3.4% Asian, 12.9% Hispanic, 1.4% Other
Age at first visit (years, *N* = 1607)	26.3% age 0–5, 27.1% age 6–10, 21.0% age 11–15, 11.4% age 16–20, 14.2% age 21+
Number of visits (*N* = 1607)	1–804, 2–329, 3–191, 4–103, 5–73, 6–39, 7–39, 8–28, 9–1
ASD by clinician (DSM5, *N* = 1592)	39.5%
ASD by SCQ (*N* = 1187)	61% no ASD, 39% ASD
ASD by SRS (*N* = 1061)	16% no ASD, 42% mild ASD, 42% severe ASD
Intellectual disability (*N* = 1536)	7.7% normal, 15.4% DD, 7.4% borderline, 23.2% mild, 38.7% mod, 7.2% severe, 0.3% profound
Current or past seizures (*N* = 1607)	12%
Current or past sleep apnea (*N* = 1323)	16.9%

* *N is less than total based on missing data for some items, and the SCQ and SRS were filled out separately from the Clinician forms and not collected on all individuals*.

**Figure 1:**
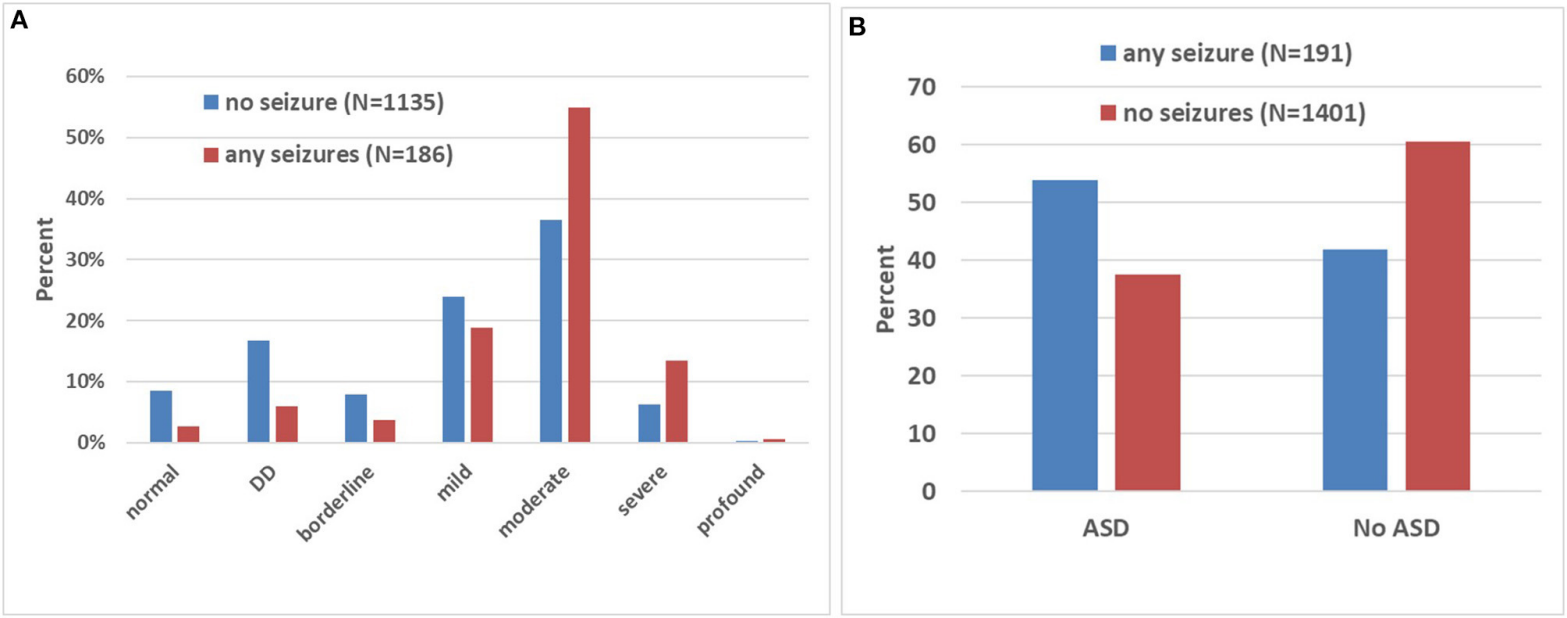
Association between intellectual disability (ID), autism spectrum disorder (ASD), and seizures in FXS. **(A)** The bar graph depicts the proportion (in percentages) of individuals with and without seizures at each level of ID. Note the higher proportion of patients with seizures at the higher levels of ID severity. DD corresponds to developmental delay in younger individuals. **(B)** The graph shows the proportion (in percentages) of individuals with and without seizures diagnosed (or not) with ASD. Note in the group with ASD, a higher proportion of patients with seizures.

In the entire cohort, there were a higher proportion of males with FXS relative to females who had current or past seizures at any time prior to baseline or during follow-up, 13.7 and 6.2%, respectively (Fisher's exact *p* < 0.0001). Seizures occurred more often in severely affected patients, with 68.7% of patients with seizures having moderate, severe, or profound ID, while only 43% of patients without seizures had this level of ID (chi-square *p* < 0.0001 for distribution of ID within the seizure and no seizure groups, Table 2, Figure 1A). Those with seizures had later onset of spoken language (age 2.37 vs. 2.02 years, ANOVA *p* = 0.03). Those with seizures were more likely to have an ASD diagnosis compared to those without seizures (53.9 vs. 41.9%, chi-square *p* < 0.0001, Table 2, Figure 1B). The group with seizures had a trend to have an increased likelihood of a diagnosis of ASD by SCQ (score of over 15) at the baseline FORWARD visit (45.9 vs. 38.3%, Fisher's exact *p* = 0.087), although not statistically significant, and increased severity of ASD symptoms by SRS-2 (9.2 vs. 16.4% absence of ASD, 53.8 vs. 40.5% severe ASD, chi-square *p* = 0.008). Current symptoms of obstructive sleep apnea were seen more often in those with seizures (4.2 vs. 2.5%) which led to a chi-square of *p* = 0.001 for the distribution of sleep apnea category between those with and without seizures. However, if one considered past history of sleep apnea, the distribution of seizures was similar in those with and without sleep apnea, making it unclear whether there is a true association. There were no effects of race/ethnicity, place of residence, household income, or level of education of parents on the likelihood of patients manifesting seizures at any time.

**Table 2:** Association of cohort characteristics with seizures.

**Characteristic**	**Seizures (*N* = 193)**	**No seizures (*N* = 1,414)**	*p*-value^**^
	***N*** **(% of total with seizures or no seizures)**	
Sex			<0.0001
Male	170 (88.1)	1067 (75.4)	
Female	23 (11.9)	347 (24.6)	
Age at first visit^*^	13.7 (8.7)	11.6 (9.4)	0.004
Age at last visit^*^	16.1 (8.9)	13.5 (9.8)	<0.00001
**Race/ethnicity**
White non-Hispanic	139 (72.0)	1061 (75.0)	0.6
Black non-Hispanic	17 (8.8)	106 (7.5)	
Asian	9 (4.7)	45 (3.2)	
Hispanic	24 (12.4)	184 (13.0)	
Other	4 (2.1)	18 (1.3)	
ASD diagnosis			<0.00001
No	80 (41.9)	847 (60.5)	
Yes	103 (53.9)	526 (37.5)	
ASD by SCQ			0.087
No	80 (54.1)	641 (61.7)	
Yes	68 (45.9)	398 (38.3)	
Unknown	8 (4.2)	28 (2)	
ASD by SRS-2			0.008
Absence of ASD	12 (9.2)	153 (16.4)	
Mild ASD	48 (36.9)	401 (43.1)	
Severe ASD	70 (48)	377 (40.5)	
Level of ID			<0.00001
None	5 (2.7)	114 (3.0)	
Devel Delay	11 (5.9)	226 (16.7)	
Borderline	7 (3.8)	107 (7.9)	
Mild	35 (18.8)	322 (23.9)	
Moderate	102 (54.8)	493 (36.5)	
Severe	25 (13.4)	85 (6.3)	
Profound	1 (0.5)	3 (0.2)	
Sleep apnea			0.001
Currently	7 (4.2)	29 (2.5)	
Past	7 (4.2)	69 (6.0)	
No	130 (78.8)	1000 (86.4)	
Don't know	21 (12.7)	60 (5.2)	

* *Values reported are mean (SD)*.

** *Significance (p-value) is reported for distribution of categories (chi-squared) in characteristic and indicates whether these are differently distributed between the group with seizures and those without*.

Patients were more likely to have reported seizures if they were older at the first and last evaluations (chi-square *p* < 0.0001 for age distribution between those with and without seizures), thought likely to be related to being past the typical age of onset of seizures in FXS. There was no effect of the number of evaluations in FORWARD.

**Figure 2:**
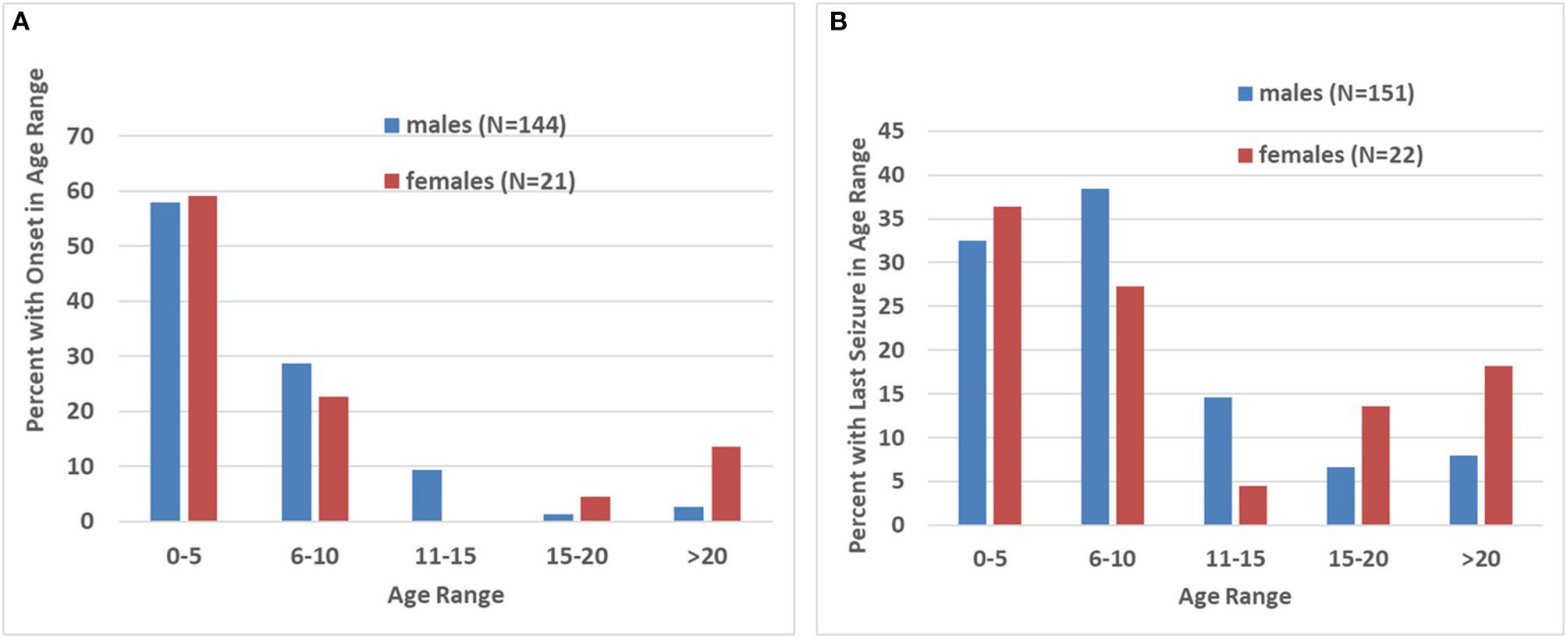
Age of onset and resolution of seizures. **(A)** Bar graph showing the proportion (in percentages) of individuals, divided by sex, with onset of seizures at different 5 year bins. Note that, with exception of a subset of females with onset after age 15, most patients with FXS displayed an onset before age 10. **(B)** Bar graph showing the proportion (in percentages) of individuals, divided by sex, experiencing last seizures at different 5 year bins. As for seizure onset, most patients had their last seizure before age 15, with exception of a subset of females who has seizure resolution after this age.

In the group of 193 patients with seizures (Table 3), the mean age at the baseline visit was 13.7 ± 8.7 and the number of evaluations during follow-up was 2.5 ± 1.8. The mean age of seizure onset was 6.4 ± 6.1, and the mean time elapsed since the last seizure was 6.4 ± 7.7 years. In most cases, seizure onset occurred before the age of 10, including 86.7 and 81.8% of males and females, respectively (Figure 2A). For males (*N* = 170), 96% had the first seizure by age 15, with only 4% presenting after age 15. For females (*N* = 23), 18.2% had their first seizure after age 15, a trend in age distribution with borderline significance (*p* = 0.058). The age of the last seizure followed a similar distribution to age of onset of seizures with 70.9% and 63.6% of seizures resolving by age 10 in males and females, respectively (Figure 2B). Only 7.9% of males and 18.2% of females had seizures continuing after age 20; this sex difference, based on a low number of females, was not significant.

**Table 3:** Characteristics of the FXS FORWARD participants with seizures.

**Sex**		**# of evaluations (clinician form)**	**Age at first evaluation**	**Age of seizure onset**	**SCQ Total Score**	**Total SRS T Scores**	**# of unique medications reported across all visits**	**Years from last seizure onset to last evaluation**
1 Male	Mean	2.48	13.39	6.22	16.17	76.12	0.95	6.42
	*N*	170	170	150	131	114	170	151
	SD	1.860	8.748	5.690	6.450	11.161	1.010	7.647
2 Female	Mean	2.35	15.83	7.36	11.94^*^	75.69	0.61	6.23
	*N*	23	23	22	17	16	23	22
	SD	1.402	8.072	8.255	4.905	13.489	0.941	7.994
Total	Mean	2.47	13.68	6.37	15.68	76.07	0.91	6.39
	*N*	193	193	172	148	130	193	173
	SD	1.809	8.686	6.060	6.422	11.415	1.006	7.668

* *p = 0.01, no other characteristics are significantly different between males and females*.

**Figure 3:**
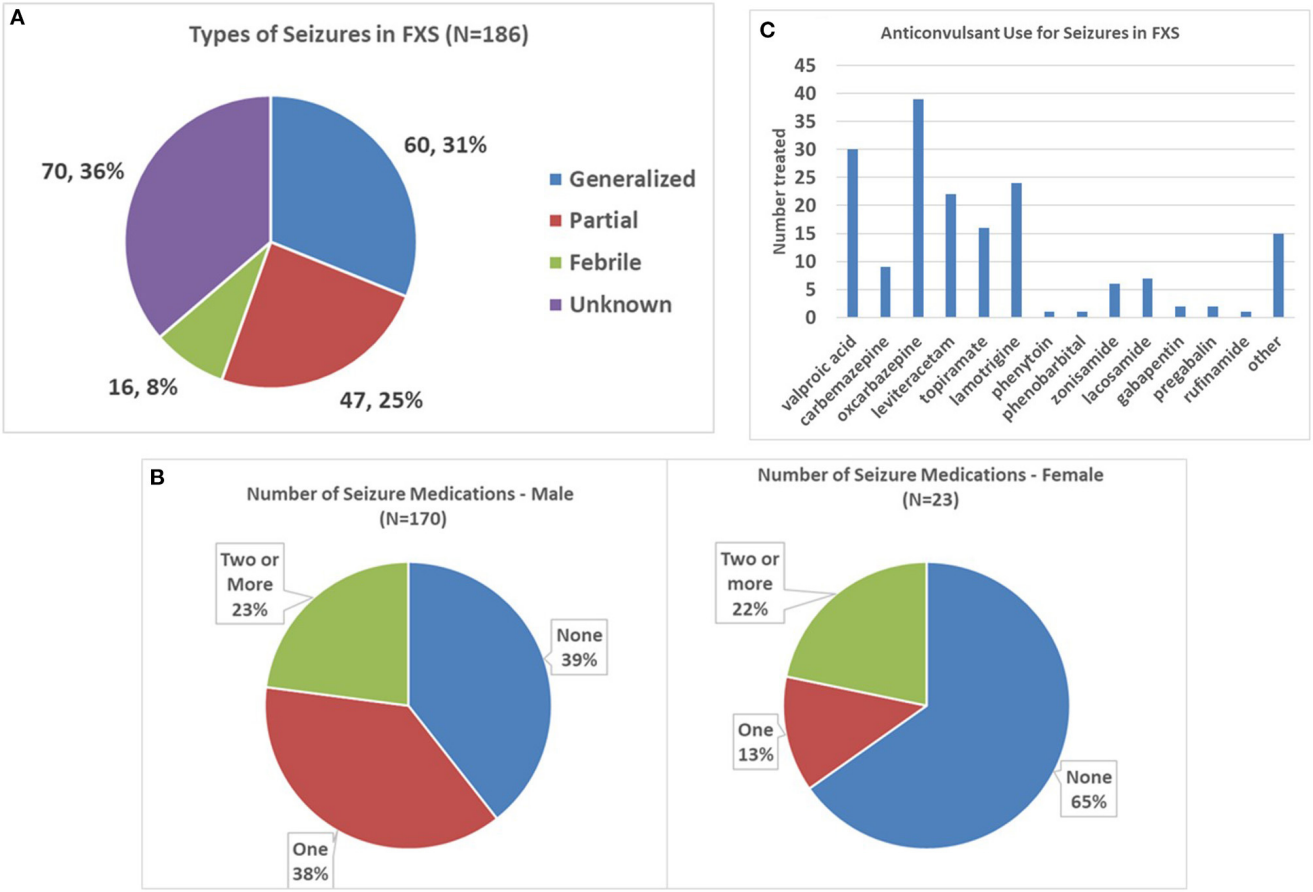
Seizure types and anticonvulsant use in FXS. **(A)** Pie graph depicting the proportion of each seizure type in the entire FXS cohort. **(B)** Graphs showing the number of anticonvulsants per patient in males and females, respectively. Note the higher proportion of males on anticonvulsants. **(C)** Number of patients with FXS using the most common anticonvulsants. Oxcarbazepine, valproic acid, and lamotrigine were the three most commonly prescribed anticonvulsants.

Partial seizures were reported in 25% and generalized seizures in 31% of patients, with febrile seizures in 8% and the remainder of seizures being of unknown type (Figure 3A). Males and females did not show a different distribution of seizure types.

The average number of anticonvulsants used across the entire group with seizures was 0.91 ± 1.01. Anticonvulsant use was reported in 60.6% of males with seizures at any evaluation vs. 34.8% of females (Fisher's exact *p* = 0.024, Figure 3B), with 37.6% of males vs. 13.0% of females requiring a single medication (chi-square *p* = 0.035), and 23 and 22%, respectively, requiring two or more medications. The most commonly used anticonvulsant was oxcarbazepine, followed by valproic acid, lamotrigine, and levetiracetam (Figure 3C).

**Figure 4:**
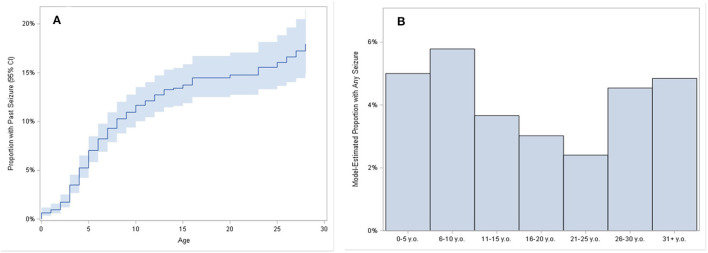
Plot of time to first seizure. **(A)** Reverse Kaplan–Meier estimates show the estimated proportion of subjects who had their first seizure onset reported, over ages 0 to 30. Band shows 95% confidence intervals for estimates. For example, approximately 7% of subjects had first seizure onset by age 5, approximately 12% of subjects had first seizure onset by age 10, and approximately 14% of subjects had first seizure onset by age 15. Bar chart of the model-estimated proportion of having any seizures during selected age periods **(B)**. Proportion estimates are derived from mixed-effect logistic regression models fit for the odds of having experienced a seizure, with categorized age as the predictor, and random intercepts for subject.

During the follow-up period, 26 incident seizures were reported in 20 males and 6 females in 1945 patient-years of follow-up, for a calculated rate of 0.013 new seizures per patient year of follow-up. Incident seizures had an age and sex distribution resembling that for age and sex distribution of seizures at baseline. Twelve males experienced a change in seizure type in 283 patient years of follow-up, indicating that seizure type changes about 4.2% of time in follow-up. Figure 4A shows a Kaplan–Meier plot of age of seizure onset in the FXS cohort, which reached a plateau by age 15 years. There was also an apparent increase in seizure onset after age 25, despite the low actual numbers of adults with seizures. This is most likely because the Kaplan–Meier model estimated the proportion based only on the subjects observed until later ages, even though many of the younger subjects would have likely reached age 25 years without having a seizure. For that reason, the Kaplan–Meier estimate of the total proportion experiencing seizures (about 17%) is greater than the raw calculation of the proportion reporting seizures (12% as noted above). Figure 4B shows the proportion of patients having seizures separated into 5 year groups. As expected, the proportions are higher in the youngest groups, tapering off until reaching the 26–30 year age group.

Patients with persistent seizures, operationally defined as those with seizures lasting over 3 years (*N* = 75), were compared with those with seizures lasting <3 years (*N* = 70) and those without seizures who had reached age 15 (*N* = 514) (Table 4). The group without seizures was limited to those over 15 years to ensure that the comparator group had a very low likelihood of developing seizures. Those without seizures were older at the first and last evaluation than the other groups; however, this was expected given the age limitation on this group. There was no significant difference in age at evaluation between the two seizure groups. Relative to those with seizures <3 years, those with seizures lasting >3 years were on more anticonvulsants (*p* < 0.0001 both first and last evaluations), were more likely to have partial seizures and less likely to have febrile seizures (*p* = 0.005 at first evaluation and 0.03 at last evaluation), made less language progress during follow-up, had more severe language impairment at the most recent visit (*p* = 0.03), and had a higher proportion of severe/profound ID (although this was not statistically significant, *p* = 0.14), but did not have higher prevalence of ASD, sleep apnea, or severity of behavioral issues on any subscale of the ABC_FX_. The lack of language progress in the group with seizures lasting >3 years was remarkable, with 25% non-verbal at the initial visit and 21.4% remaining non-verbal at the last visit compared to 14.4% non-verbal at the initial visit and 5.7% at the last visit for those with seizures lasting <3 years. Underscoring the phenotypical severity associated with having seizures regardless of duration, those with no seizures had significantly less severe ID than the <3 year seizure group (*p* = 0.003), less severe language impairment at the first but not the last visit for those with seizures <3 years (*p* = 0.038 and 0.15, respectively), less severe language impairment than those with seizures >3 years at both visits (*p* = 0.0004 and 0.0062, respectively), a higher percent with sustained conversation ability at both visits than those with seizures <3 years at both visits (*p* = 0.0003 and 0.01, respectively), a lower proportion with ASD diagnosis than both seizure groups at both visits (*p* = 0.009 for both groups at the last visit), and lower scores on the ABC_FX_ Hyperactivity, Irritability, and Stereotypy subscales than both seizure groups at both visits (*p*-values ranging from < 0.0001 to 0.029).

**Table 4:** Comparison of characteristics between groups with no seizures, seizures reported for < 3 years, and seizures reported for > 3 years^*^.

	**Overall (*****N*** **=** **659)**		**No Seizures (*****N*** **=** **514)**		**Seizures** **<** **3 years (*****N*** **=** **70)**		**Seizures** **>** **3 years (*****N*** **=** **75)**		***p*** **-values**			
**Variable**	***n***	**% or Mean (SD)**	***n***	**% or Mean (SD)**	***n***	**% or Mean (SD)**	***n***	**% or Mean (SD)**	**Overall**	**1 vs. 2**	**1 vs. 3**	**2 vs. 3**
Number of evaluations	659	2.8 (2.0)	514	2.8 (2.0)	70	2.6 (1.7)	75	2.8 (2.1)	0.7533	0.4488	0.8982	0.6178
Number of evaluations [Min, Max]	659	(1, 9)	514	(1, 9)	70	(1, 8)	75	(1, 8)				
Number of evaluations–binary									0.5789	0.8537	0.3185	0.3821
1	235	35.7%	180	35.0%	24	34.3%	31	41.3%				
2+	424	64.3%	334	65.0%	46	65.7%	44	58.7%				
Gender									0.0535	0.0537	0.1033	0.7522
Male	520	79.0%	395	77.0%	61	87.1%	64	85.3%				
Female	138	21.0%	118	23.0%	9	12.9%	11	14.7%				
Age at first evaluation	659	19.9 (9.6)	514	21.1 (9.4)	70	14.7 (8.3)	75	16.4 (9.5)	<0.0001	<0.0001	<0.0001	0.2669
Age at last evaluation	659	22.6 (9.3)	514	23.8 (9.0)	70	17.6 (8.4)	75	18.9 (9.6)	<0.0001	<0.0001	<0.0001	0.3685
Years between first and last evaluation	419	4.3 (2.2)	330	4.3 (2.3)	46	4.3 (2.1)	43	4.4 (2.2)	0.9798	0.8835	0.4309	0.4973
Age of onset	135	6.7 (6.6)		N/A	66	5.9 (5.3)	69	7.5 (7.6)	N/A	N/A	N/A	0.1625
Mean inter-evaluation period	423	1.8 (1.2)	333	1.8 (1.2)	46	2.0 (1.2)	44	1.6 (1.0)	0.3655	0.3427	0.3576	0.1326
Number of AEDs first evaluation	659	0.1 (0.5)	514	0.0 (0.0)	70	0.3 (0.5)	75	1.1 (1.0)	<0.0001	<0.0001	<0.0001	<0.0001
Number of AEDs first evaluation [Median (IQR)]	659	0.0 (0.0-0.0)	514	0.0 (0.0-0.0)	70	0.0 (0.0-0.0)	75	1.0 (0.0-2.0)				
Number of AEDs last evaluation	659	0.2 (0.5)	514	0.0 (0.0)	70	0.3 (0.5)	75	1.3 (1.0)	<0.0001	<0.0001	<0.0001	<0.0001
Number of AEDs last evaluation [Median (IQR)]	659	0.0 (0.0-0.0)	514	0.0 (0.0-0.0)	70	0.0 (0.0-0.0)	75	1.0 (1.0-2.0)				
Seizure type first seizure evaluation									N/A	N/A	N/A	0.0051
Generalized	61	45.5%		N/A	28	43.8%	33	47.1%				
Partial	46	34.3%		N/A	16	25.0%	30	42.9%				
Febrile	11	8.2%		N/A	10	15.6%	1	1.4%				
Unknown	16	11.9%		N/A	10	15.6%	6	8.6%				
Seizure type last seizure evaluation									N/A	N/A	N/A	0.0363
Generalized	39	45.9%		N/A	19	42.2%	20	50.0%				
Partial	25	29.4%		N/A	10	22.2%	15	37.5%				
Febrile	11	12.9%		N/A	10	22.2%	1	2.5%				
Unknown	10	11.8%		N/A	6	13.3%	4	10.0%				
Intellectual disability Dx last evaluation									0.0001	0.0003	0.0735	0.1380
No ID	35	5.5%	31	6.3%	4	5.8%	0	0.0%				
Delayed development	3	0.5%	0	0.0%	3	4.3%	0	0.0%				
Borderline	35	5.5%	30	6.1%	2	2.9%	3	4.3%				
Mild	146	23.0%	119	24.0%	11	15.9%	16	22.9%				
Moderate	356	56.2%	274	55.4%	42	60.9%	40	57.1%				
Severe	57	9.0%	40	8.1%	7	10.1%	10	14.3%				
Profound	2	0.3%	1	0.2%	0	0.0%	1	1.4%				
Language milestone (Q24) first evaluation									0.0049	0.0378	0.0004	0.6416
Non-verbal	12	1.8%	6	1.2%	2	2.9%	4	5.3%				
Signing	7	1.1%	5	1.0%	0	0.0%	2	2.7%				
Babbling	2	0.3%	0	0.0%	1	1.4%	1	1.3%				
Few words	52	8.0%	34	6.7%	7	10.1%	11	14.7%				
Word combinations	64	9.9%	47	9.3%	9	13.0%	8	10.7%				
Phrases	512	78.9%	413	81.8%	50	72.5%	49	65.3%				
Sustained conversation (Q26) first evaluation									0.0009	0.0774	0.0003	0.5314
Non-verbal	26	4.1%	15	3.0%	4	6.0%	7	9.6%				
Yes, sustained conversion	385	60.3%	320	64.1%	34	50.7%	31	42.5%				
No sustained conversion	228	35.7%	164	32.9%	29	43.3%	35	47.9%				
Language milestone (Q24) last evaluation									0.0034	0.1536	0.0062	0.0358
Non-verbal	8	1.2%	5	1.0%	1	1.4%	2	2.7%				
Signing	8	1.2%	5	1.0%	2	2.9%	1	1.3%				
Babbling	4	0.6%	2	0.4%	0	0.0%	2	2.7%				
Few words	40	6.1%	28	5.5%	1	1.4%	11	14.7%				
Word combinations	53	8.1%	37	7.3%	10	14.3%	6	8.0%				
Phrases	538	82.6%	429	84.8%	56	80.0%	53	70.7%				
Sustained conversation (Q26) last evaluation									0.0236	0.2432	0.0122	0.1287
Non-verbal	12	2.0%	9	1.9%	0	0.0%	3	4.5%				
Yes, sustained conversion	404	65.8%	329	68.4%	41	62.1%	34	50.7%				
No sustained conversion	198	32.2%	143	29.7%	25	37.9%	30	44.8%				
Autism Dx ever									0.0003	0.0005	0.0246	0.1952
No	321	48.9%	273	53.2%	20	28.6%	28	37.8%				
Yes	328	49.9%	234	45.6%	48	68.6%	46	62.2%				
Autism Dx current (last evaluation)									0.0022	0.0095	0.0094	0.3945
Yes	276	43.0%	196	39.0%	39	57.4%	41	57.7%				
No	331	51.6%	277	55.1%	28	41.2%	26	36.6%				
Don't know	35	5.5%	30	6.0%	1	1.5%	4	5.6%				
Sleep apnea last evaluation									0.1434	0.3075	0.0555	0.6080
No	378	97.2%	295	98.0%	43	95.6%	40	93.0%				
Yes	11	2.8%	6	2.0%	2	4.4%	3	7.0%				
ABC hyperactivity first evaluation	506	18.8 (7.4)	393	17.9 (6.9)	58	23.3 (8.1)	55	20.6 (8.0)	<0.0001	<0.0001	0.0076	0.0795
ABC inappropriate Speech first evaluation	506	8.4 (3.4)	393	8.3 (3.4)	58	9.1 (3.6)	55	8.1 (3.3)	0.2196	0.1187	0.5732	0.1172
ABC irritability first evaluation	506	31.0 (11.9)	393	29.9 (11.4)	58	35.8 (14.1)	55	33.2 (11.8)	0.0006	0.0004	0.0473	0.2830
ABC social avoidance first evaluation	506	7.7 (3.4)	393	7.7 (3.4)	58	7.6 (3.4)	55	7.4 (3.4)	0.7930	0.8768	0.4987	0.6881
ABC social unresponsiveness first evaluation	506	18.8 (5.7)	393	18.5 (5.9)	58	19.9 (5.3)	55	19.6 (5.3)	0.1154	0.0811	0.1916	0.7393
ABC stereotypy first evaluation	506	10.9 (4.8)	393	10.5 (4.6)	58	12.7 (5.4)	55	11.9 (5.1)	0.0011	0.0010	0.0296	0.4655
ABC hyperactivity last evaluation	504	17.5 (6.9)	382	16.4 (6.2)	58	21.5 (7.2)	64	20.1 (8.3)	<0.0001	<0.0001	<0.0001	0.3237
ABC inappropriate Speech last evaluation	504	8.0 (3.4)	382	7.8 (3.4)	58	8.7 (3.5)	64	8.4 (3.4)	0.1310	0.0789	0.2204	0.6524
ABC irritability last evaluation	504	30.0 (11.7)	382	28.8 (11.1)	58	33.5 (12.3)	64	34.1 (13.0)	0.0002	0.0032	0.0007	0.8072
ABC social avoidance last evaluation	504	7.5 (3.4)	382	7.4 (3.3)	58	8.1 (3.8)	64	7.2 (3.3)	0.2760	0.1532	0.6003	0.1568
ABC social unresponsiveness last evaluation	504	18.4 (5.7)	382	18.2 (5.8)	58	19.0 (5.1)	64	19.5 (5.1)	0.1634	0.3221	0.0856	0.5669
ABC stereotypy last evaluation	504	10.5 (4.6)	382	10.1 (4.3)	58	11.8 (5.0)	64	12.0 (5.0)	0.0006	0.0061	0.0016	0.8347

* *for the p-values listed for group comparisons, 1 = no seizures, 2 = seizures for <3 years, 3 = seizures for > 3 years*.

## Discussion

In this study, we characterized seizures in the largest clinically evaluated cohort of individuals with FXS. In addition, this is the only longitudinal study of seizures to date in FXS and thus the only study ever to evaluate incident onset of seizures prospectively in this syndrome. In addition to confirming previously reported characteristics of individuals with FXS and seizures, such as prevalence, sex distribution (13.7% males, 6.2% females), and association with ASD (19, 26, 33), we identified an association with current sleep apnea and additional features. Those with seizures were also more likely to have more severe ID and later onset of expressive language, findings which have not been reported before, likely due to lack of sufficient numbers of subjects (10–15, 26) or lack of clinical assessment or evaluation of levels of ID and language (19, 33) in prior studies. The group with seizures was also more likely to have more severe behavioral problems, as measured by the ABC_FX_ (Hyperactivity, Irritability, and Stereotypy subscales). Availability of longitudinal data allowed the delineation of key aspects in the evolution of seizures in FXS and their association with other features of the disorder. A longer course of epilepsy in FXS was associated with greater overall cognitive and language impairments, but not more severe behavioral problems.

In this FORWARD cohort, individuals with FXS and seizures were more likely to have ASD diagnosed by the clinician based on DSM5 criteria. The association between seizures and a clinical diagnosis of ASD in children with FXS is supported by the finding that this subset has more severe ASD symptoms on the SCQ and SRS-2. Consistent with our findings, previous studies have found an association between the diagnosis of ASD and seizures and children with ASD with seizures have been found to have more severe ASD symptoms (34, 35). However, although previous studies suggested that children with ASD and genetic disorders have high rates of epilepsy (36), this study suggests that the rate of seizures in FXS is rather moderate, at 12%, comparable to the 12.1% rate of epilepsy in individuals with idiopathic ASD reported in a recent meta-analysis (37). Additionally, studies on children with ASD also demonstrate that many have subclinical epileptiform discharges in a centrotemporal pattern (38, 39) and occipital regions (40), similar to EEG profiles reported in FXS (13, 15, 19–21). Children with ASD and EEGs with subclinical epileptiform discharges are found to be more severely impaired (35, 41) but may show improvement in behavior and cognition with treatments targeted to reduced epileptiform discharges (35, 38, 42). Although EEG data were not collected in the FORWARD study, children with FXS and an abnormal EEG are a subgroup of FXS patients that may warrant closer study.

Onset of seizures in FXS was typically (>80%) below age 10, with a very low proportion of males having seizure onset after age 15 years. A higher percent of females had seizure onset after age 15 years, which suggests a slightly different pattern for seizure onset in females, although females with first seizure at age >15 represented only four patients. Thus, the marginally significant chi-square (*p* = 0.058) for a different male–female distribution of age of seizure onset may be due to the relatively small number of females with seizures in the cohort. Consequently, a larger cohort would be needed to confirm that females with FXS have a larger proportion of late-onset seizures than males. There is a new concern in adolescents and adults with idiopathic ASD of having new-onset seizures (43), especially in those with ID (44), which appears to be linked to increased morbidity (45, 46). Interestingly, we did not find this trend in those with FXS, making this less of a concern in this genetic disorder.

As with idiopathic ASD and many other ID syndromes, seizure type was variable between patients. However, the proportions of generalized and partial seizures are in general agreement with the literature (13–15, 19). Overall, the number of anticonvulsants required for those with seizures was low, consistent with past observations (15, 19). As might be expected considering that males with FXS are more affected than females, males with seizures were more likely to be on anticonvulsants, including multiple drugs, and to be followed in clinic and in FORWARD longer than females with seizures. The anticonvulsants used in the FXS population were consistent with recommendations in the field (2, 19, 47) and were those typically employed in any pediatric seizure population, and similar perhaps with somewhat less use of levetiracetam, most likely due to concerns about aggravating behavior.

For all participants with follow-up visits in FORWARD, new (i.e., incident) seizures were rare, with risk being only approximately 1% per patient year of follow-up in FORWARD. Risk of change in seizure type was only approximately 4% per patient year. The Kaplan–Meier plot (Figure 4) shows risk of having seizures at any given age across the lifespan. It is clear that most seizures are present early in life. After being stable through the 15–25 year age period with fewer patients having any seizure during this time period, the proportion of seizures goes up after age 25, most likely due to the small number of adults >25 years old included in FORWARD to date. There is also a likely referral bias such that patients are more likely to remain in care at an FXS clinic if they have ongoing seizures or be referred to an FXS clinic if they have new-onset seizures. This may be particularly the case for clinics run by pediatric neurologists, who tend to see adults with FXS more readily. Since the FORWARD project is now focused on increasing enrollment of adults, it is expected that more definitive estimates can be obtained for seizure prevalence and onset in adults upon future analysis of a larger cohort.

When patients with longer duration of seizures (>3 years) were compared with those with shorter duration (<3 years), only a few differences emerged, including increased use of anticonvulsants, higher prevalence of partial seizures, and lower prevalence of febrile seizures and, most importantly, more severe language outcomes despite lack of differences in behavioral abnormalities (including ASD) and non-significant increases in ID severity. This suggests that a longer course of seizures in FXS is an index of severity linked to more severe outcome in this some functional domains. It is possible that the division of the groups at 3 years of persistent seizures did not isolate those with the most severe phenotypes and a 5 year cutoff might have been better to more fully explore this concept. Nonetheless, there were not enough patients in FORWARD with 5 years of longitudinal data at present to support such an analysis.

Strengths of this study are the size of the subject sample, very large for rare disease standards, and the ability to examine longitudinal data. Weaknesses include lack of availability of follow-up visits into the late teenage years in about half the cohort, lack of complete evaluations of seizure type (many were undefined), and a relatively small population of adults in the current FORWARD database. Additionally, as participants with FXS were not a randomly selected, sampling biases inherent to clinic-based samples cannot be discounted.

Based on the data from this study, the prototypical individual with FXS and seizures would be male, with moderate or severe ID and expressive language delay, with a DSM-5 diagnosis of ASD and a corresponding SCQ >15 and SRS-2 in the severe range, who had a first evaluation at a FORWARD (perhaps any FXS) clinic after age 5. Females with FXS and seizures would show similar characteristics; however, among females a subgroup with a distinctive but uncommon profile of onset of seizures after age 15 is identified. It appears that this subgroup has greater cognitive impairment than other females with or without seizures and had a first evaluation at a FORWARD clinic after age 15. The duration of seizures also seems to be a parameter associated with worse outcomes, particularly in cognitive function.

FMRP has been linked to seizures in FXS through excessive mGluR5 signaling of dendritic translation, based on reversal of audiogenic seizures by mGluR5 blockers in the fmr1 knockout mouse (48). FMRP also operates presynaptically and may cause seizures through interruption of a direct interaction of FMRP with a presynaptic BK channel subunit (49). Seizures are associated with mutations in CYFIP2, which interacts with FMRP (50), and FMRP appears to underlie enhanced mLTD in adult rats triggered by early-life seizures (51). Thus, absence of FMRP may potentiate seizure *via* multiple neural mechanisms which are likely to vary between patients, thus resulting in variable penetrance and severity of seizures in FXS. However, likely the presence of seizures is a signal of more problematic neural and synaptic dysfunction related to variation in FMRP deficits in cells, and variation in these multiple interacting pathways. The present study adds to the understanding of the characteristics, risk factors, and course of seizures in FXS and provides a basis for anticipatory guidance for clinicians and families. The study also defines gaps and additional areas of investigation that could answer further questions about seizures, their neural underpinnings, and their evolution across the lifespan in FXS.

## Data Availability Statement

The raw data supporting the conclusions of this article will be made available by the authors, without undue reservation.

## Ethics Statement

The studies involving human participants were reviewed and approved by Institutional IRBs at all FORWARD academic sites. Written informed consent to participate in this study was provided by the participant's legal guardian/next of kin.

## Forward Consortium

Elizabeth Berry-Kravis (Rush University Medical Center), Milen Velinov (Rutgers Robert Wood Johnson Medical School), Amy L. Talboy (Emory University School of Medicine), Stephanie L. Sherman (Emory University School of Medicine), Walter E. Kaufmann (Emory University School of Medicine), Marcy Schuster (Elwyn Inc), Nicole Tartaglia (Children's Hospital Colorado), Robyn A. Filipink (University of Pittsburgh School of Medicine), Dejan B. Budimirovic (Kennedy Krieger Institute, Johns Hopkins Medical Institutions), Deborah Barbouth (University of Miami, Miller School of Medicine), Amy Lightbody (Stanford University School of Medicine), Allan Reiss (Stanford University School of Medicine), Carol M. Delahunty (The Metrohealth System, Cleveland), Randi J. Hagerman (MIND Institute, University of California Davis Medical Center), David Hessl (MIND Institute, University of California Davis Medical Center), Craig A. Erickson (Cincinnati Children's Hospital Medical Center), Gary Feldman (Miller Children's & Woman's Hospital), Jonathan D. Picker (Boston Children's Hospital), Ave M. Lachiewicz (Duke Health Center, Lenox Baker Children's Hospital), Holly K. Harris (Baylor College of Medicine & Meyer Center for Developmental Pediatrics), Amy Esler (University of Minnesota), Richard E. Frye (Barrow Neurological Institute at Phoenix Children's Hospital), Patricia A. Evans (University of Texas Southwestern Medical Center), Mary Ann Morris (University of Texas Southwestern Medical Center), Barbara A. Haas-Givler (Geisinger's Autism & Developmental Medicine Institute), Andrea L. Gropman (Children's National Medical Center), Ryan S. Uy (Children's National Medical Center), Reymundo Lozano (Icahn School of Medicine at Mount Sinai), Carrie Buchanan (Greenwood Genetic Center), Jean A. Frazier (Eunice Kennedy Shriver Center, University of Massachusetts Medical School), Stephanie M. Morris (Washington University in St. Louis).

## Author Contributions

EB-K, RFi, RFr, SG, SM, HA, T-HC, and WK: study concept and design, acquisition, analysis, and interpretation of data, drafting and critical revision of the manuscript, and have approved it for publication. All authors contributed to the article and approved the submitted version.

## Funding

Work on this publication was supported by cooperative agreements (U01DD000231, U19DD000753, and U01DD001189), funded by the Centers for Disease Control and Prevention.

## Author Disclaimer

Its contents are solely the responsibility of the authors and do not necessarily represent the official views of the Centers for Disease Control and Prevention or the Department of Health and Human Services.

## Conflict of Interest

The authors declare that the research was conducted in the absence of any commercial or financial relationships that could be construed as a potential conflict of interest.

## Publisher's Note

All claims expressed in this article are solely those of the authors and do not necessarily represent those of their affiliated organizations, or those of the publisher, the editors and the reviewers. Any product that may be evaluated in this article, or claim that may be made by its manufacturer, is not guaranteed or endorsed by the publisher.
